# Floquet–Bloch solutions in a sawtooth photonic crystal

**DOI:** 10.1007/s11082-017-0939-1

**Published:** 2017-02-21

**Authors:** S. Caffrey, G. V. Morozov, D. W. L. Sprung, J. Martorell

**Affiliations:** 1 Scottish Universities Physics Alliance (SUPA), Institute of Thin Films, Sensors and Imaging, University of the West of Scotland, Paisley, PA1 2BE Scotland, UK; 2 Department of Physics and Astronomy, McMaster University, Hamilton, Ontario L8S 4M1 Canada; 3 Departament de Fisica Quantica i Astrofisica, Facultat de Fisica, University of Barcelona, 08028 Barcelona, Spain

**Keywords:** Sawtooth photonic crystal, Band structure, Floquet–Bloch solutions

## Abstract

Band structure of a sawtooth photonic crystal for optical wave propagation along the axis of periodicity is investigated. Floquet–Bloch solutions are found and illustrated for the bandgaps, allowed bands, and bandedges of the crystal. Special attention is given to the cases where Floquet–Bloch solutions become periodic functions.

## Introduction

**Fig. 1:**
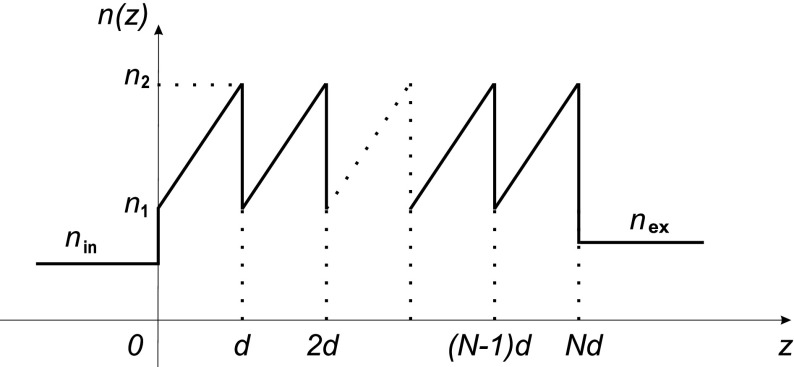
A sawtooth photonic crystal with the refractive index increasing linearly from $n_1$ to $n_2$ inside each period of width *d*; *N* is the number of periods; $n_{\mathrm{in}}$ and $n_{\mathrm{ex}}$ are the refractive indices of the incident and exit media respectively

In a recent paper (Morozov et al. 2013), we presented solutions for optical wave propagation through a sawtooth crystal, i.e. a one-dimensional (1D) photonic crystal constructed of layers with a linearly increasing refractive index *n*(*z*) in each period, see Fig. 1, in the exact analytical form. In particular, we expressed the fields inside the crystal in terms of the normalized solutions $u(z)\,[u(0)=1,u'(0)=0]$ and $v(z)\,[v(0)=0,v'(0)=1]$, each expressed as a superposition of Bessel functions. With the aid of the transfer matrix method, the reflection/transmission characteristics of the crystal were obtained.

In this paper, we analyze the band structure of a sawtooth crystal in the case of normal propagation. It will be shown that it can be expressed in terms of just two parameters only: a period-average dimensionless wavenumber $kn_{\mathrm{av}}d$ of the light inside the crystal and the Fresnel reflection coefficient $r_{21}$ between the layers of refractive indices $n_2$ and $n_1$, i.e.

1 $$\begin{aligned} kn_{\mathrm{av}}d \equiv k\,\frac{n_2+n_1}{2}\,d, \quad r_{21}=\frac{n_2-n_1}{n_2+n_1}, \end{aligned}$$

where *k* is the wavenumber of light in vacuum. Then, we express the fields inside the crystal in terms of the Floquet–Bloch solutions and illustrate their behavior in the allowed bands, in the bandgaps, and at the bandedges respectively. Special attention is given to periodic solutions. Overall, the results add to a better understanding of the behavior of light within photonic crystals with linearly graded refractive index layers, recently studied in Morozov et al. (2013), Fernandez-Guasti and Diamant (2015), Wu et al. (2011), Rauh et al. (2010).

## Band structure of a sawtooth crystal

Propagation of linearly polarized light of vacuum wavenumber *k* along the axis of periodicity *z* of a sawtooth photonic crystal with period *d*, see Fig. 1, reduces to solving Hill’s equation

2 $$\begin{aligned} \frac{d^2E(z)}{dz^2}+k^2 n^2(z) E(z) =0, \quad n(z+d)=n(z), \end{aligned}$$

where on the first period, i.e. for $0<z<d$,

3 $$\begin{aligned} n(z) = n_1 + (n_2-n_1)\frac{z}{d}. \end{aligned}$$

The total electric field of propagating light is then

4 $$\begin{aligned} \mathbf{E} = E(z)\exp (-ikc\,t)\,\hat{\mathbf{y}}, \end{aligned}$$

where *c* is velocity of light in vacuum, and $\hat{\mathbf{y}}$ is a unit vector along the polarization direction, which is normal to the plane of incidence. In accordance with Morozov et al. (2013), the normalized solutions of Eq. (1) are expressed as

5 $$\begin{aligned} u(z) & = \frac{{\tilde{v}}'(0)}{\sqrt{\gamma }}{\tilde{u}}(z)-\frac{{\tilde{u}}'(0)}{\sqrt{\gamma }}{\tilde{v}}(z),\nonumber \\ v(z) & = -\frac{{\tilde{v}}(0)}{\sqrt{\gamma }}{\tilde{u}}(z)+\frac{{\tilde{u}}(0)}{\sqrt{\gamma }}{\tilde{v}}(z), \end{aligned}$$

with

6 $$\begin{aligned} {\tilde{u}}(z) & = \frac{\pi }{2^{1/4}\varGamma {\left( 1/4\right) }}\left[ \sqrt{\gamma }\left( z-z_0\right) \right] ^{1/2}J_{-1/4}\left[ \frac{\gamma \left( z-z_0\right) ^{2}}{4}\right] ,\nonumber \\ {\tilde{v}}(z) & = \frac{\pi }{2^{3/4}\varGamma {\left( 3/4\right) }}\left[ \sqrt{\gamma }\left( z-z_0\right) \right] ^{1/2}J_{1/4}\left[ \frac{\gamma \left( z-z_0\right) ^{2}}{4}\right] , \end{aligned}$$

where

7 $$\begin{aligned} \gamma = \frac{2k\left( n_2-n_1\right) }{d} = \frac{4kn_{\mathrm{av}}\,r_{21}}{d}, \quad z_{0} = -\frac{n_{1}d}{n_{2}-n_{1}} = -\frac{(1-r_{21})\,d}{2\,r_{21}}. \end{aligned}$$

The overall field *E*(*z*) within the crystal, which is a general solution of Eq. (2) for $0<z<Nd$, is then

8 $$\begin{aligned} E(z) = Au(z) + Bv(z), \end{aligned}$$

where the constants *A* and *B* can be found from the boundary conditions at the points $z=0$ and $z=Nd$.

In accordance with the Floquet–Bloch theory (Magnus and Winkler 2004; Eastham 1975; Stoker 1950), the overall field *E*(*z*) within a periodic crystal can be also expressed as a superposition of two Floquet–Bloch solutions, which in most cases take the form

9 $$\begin{aligned} F_{1,2}(z) = P_{1,2}(z)\, e^{\pm i \varphi z/d}, \quad P_{1,2}(z+d)=P_{1,2}(z)\,, \end{aligned}$$

where the Bloch phase $\varphi$,

10 $$\begin{aligned} 2\cos (\varphi )=u(d)+v'(d), \end{aligned}$$

defines the band structure of a periodic potential. One can see that the solutions $F_{1,2}(z)$ satisfy a translational property

11 $$\begin{aligned} F_{1,2}(z+d) = \rho _{1,2} F_{1,2}(z), \quad \rho _{1,2}=\exp (\pm i\varphi ). \end{aligned}$$

In the allowed bands the Bloch phase is real, $-1< \cos (\varphi ) < 1$, and, as a result, both Floquet–Bloch solutions are oscillating functions. In the bandgaps the Bloch phase is complex, $\left| \cos (\varphi )\right| >1$, in particular $\varphi = m \pi + i\varphi ^{''}$, where $m =1, 2, 3 \ldots , \varphi ^{''}$ is real and, as a result, one Floquet–Bloch solution decays while the other one grows along the *z*-axis of propagation. The overall field *E*(*z*) also decays in the bandgaps.

If $\cos (\varphi )={\pm }1$, two cases might occur. Typically, this condition corresponds to the boundaries between the bandgaps and allowed bands. On those boundaries the Floquet–Bloch solutions become identical periodic functions, i.e. $F_{2}(z)=F_{1}(z)\equiv F(z)$, of period *d* when $\cos (\varphi ) = 1$ ($\rho _1=\rho _2 \equiv \rho = 1$), or of period 2*d* when $\cos (\varphi ) = -1$ ($\rho _1=\rho _2 \equiv \rho = -1$). To account for this anomaly, another particular solution must be sought to complete the general solution of Eq. (2). It is known as the hybrid Floquet mode *G*(*z*)

12 $$\begin{aligned} G(z) = [P_2(z)+zP_1(z)]\,e^{i\varphi z/d}, \end{aligned}$$

and it satisfies a translational property

13 $$\begin{aligned} G(z+d)=\rho G(z) +\rho d F(z). \end{aligned}$$

An appearance of a hybrid Floquet mode was discussed in the context of 1D superlattices in Cottey (1971, 1972) and in the context of 1D photonic crystals in Morozov and Sprung (2011, 2012).

If in addition to $\cos (\varphi )=\pm 1$ ($u(d)+v'(d) = \pm 2$), the normalized solutions satisfy the condition $v(d) = u'(d) = 0$, so-called incipient bands (vanishing gaps) appear. Those are points of contact between distinct allowed bands (i.e. a special type of band crossing), where both distinct functions $F_{1,2}(z)$ and, as a result, the overall field *E*(*z*) within the crystal, become periodic, again of period *d* if $\cos (\varphi ) = 1$ ($\rho _1=\rho _2 \equiv \rho = 1$), or of period 2*d* if $\cos (\varphi ) = -1$ ($\rho _1=\rho _2 \equiv \rho = -1$). There are periodic potentials, for which vanishing gaps exist in the form of discrete points (Morozov and Sprung 2011, 2012), straight lines (Morozov and Sprung 2015), and continuous second-order curves (Caffrey et al. 2016).

For a sawtooth crystal one has

14 $$\begin{aligned} {u}(d) & =  \frac{\pi }{4\sqrt{2}}\frac{{(1-r_{21})}^{3/2}}{r_{21}}\,(1+r_{21})^{1/2}\,kn_{\mathrm{av}}d \nonumber \\&\quad \times \left[ J_{-3/4}\left( {z^{2}_{1}/4}\right) J_{-1/4}\left( {z^{2}_{2}/4}\right) +J_{3/4}\left( {z^{2}_{1}/4}\right) J_{1/4}\left( {z^{2}_{2}/4}\right) \right] ,\nonumber \\ u'(d)\,d & =  -\frac{\pi }{4\sqrt{2}}\frac{{(1-r_{21}^2)}^{3/2}}{r_{21}}\,(kn_{\mathrm{av}}d)^2 \nonumber \\&\quad \times \left[ J_{-3/4}\left( {z^{2}_{1}/4}\right) J_{3/4}\left( {z^{2}_{2}/4}\right) -J_{3/4}\left( {z^{2}_{1}/4}\right) J_{-3/4}\left( {z^{2}_{2}/4}\right) \right] ,\nonumber \\ \frac{{v}(d)}{d} & =  -\frac{\pi }{4\sqrt{2}}\frac{{(1-r_{21}^2)}^{1/2}}{r_{21}} \nonumber \\&\quad \times \left[ J_{1/4}\left( {z^{2}_{1}/4}\right) J_{-1/4}\left( {z^{2}_{2}/4}\right) +J_{-1/4}\left( {z^{2}_{1}/4}\right) J_{1/4}\left( {z^{2}_{2}/4}\right) \right] ,\nonumber \\ {v'}(d) & =  \frac{\pi }{4\sqrt{2}}\frac{{(1+r_{21})}^{3/2}}{r_{21}}\,(1-r_{21})^{1/2}\,kn_{\mathrm{av}}d \nonumber \\&\quad \times \left[ J_{1/4}\left( {z^{2}_{1}/4}\right) J_{3/4}\left( {z^{2}_{2}/4}\right) +J_{-1/4}\left( {z^{2}_{1}/4}\right) J_{-3/4}\left( {z^{2}_{2}/4}\right) \right] , \end{aligned}$$

with dimensionless parameters $z_{1}$ and $z_{2}$ defined as

15 $$\begin{aligned} z_1 & = -\sqrt{\gamma }\, z_{0} = \left( \frac{2kdn^{2}_{1}}{n_{2}-n_{1}}\right) ^{1/2} = \frac{1-r_{21}}{\sqrt{r_{21}}}\,\sqrt{kn_{\mathrm{av}}d}\,, \nonumber \\ z_2 & = \sqrt{\gamma }\, (d-z_{0}) = \left( \frac{2kdn^{2}_{2}}{n_{2}-n_{1}}\right) ^{1/2} = \frac{1+r_{21}}{\sqrt{r_{21}}}\,\sqrt{kn_{\mathrm{av}}d}\,. \end{aligned}$$

**Fig. 2:**
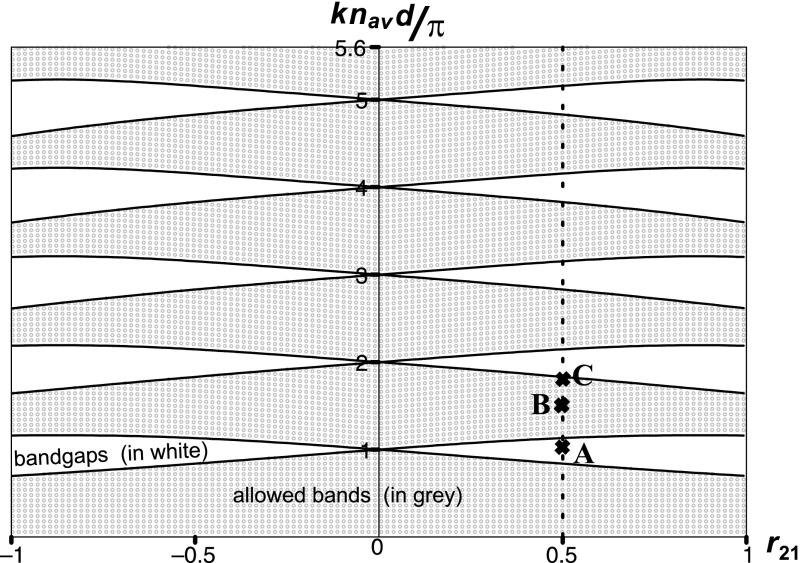
Band structure of a sawtooth photonic crystal, *k* is the vacuum wavenumber of propagating light, *d* is the period of a crystal, $n_{\mathrm{av}} = (n_1+n_2)/2, r_{21} = (n_2-n_1)/(n_2+n_1)$

One can see that the Bloch phase $\varphi$, see Eq. (10), is defined by two parameters only: $kn_{\mathrm{av}}d$ and $r_{21}$. The results are shown in Fig. 2. Since $u'(d)$ and *v*(*d*) are never zero simultaneously, a sawtooth crystal does not possess vanishing gaps, in agreement with Mogilner and Loly (1992).

**Fig. 3:**
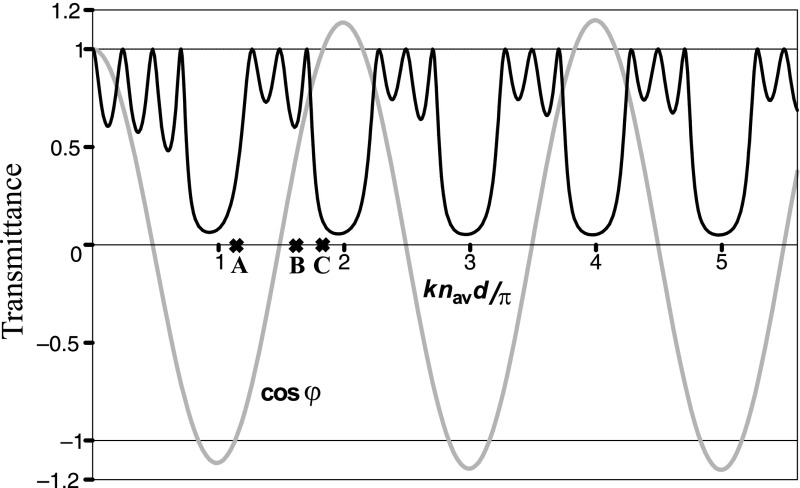
The transmittance and the Bloch phase $\cos (\varphi )$ of a sawtooth crystal with $r_{21}=0.5$ ($n_1=1.5, n_2=4.5$), $N=4$. The refractive indices of the incident and exit media are $n_{\mathrm{in}}=n_{\mathrm{ex}}=n_1$

The energy transmission coefficient (transmittance), *T*, for an optical wave of vacuum wavenumber *k* impinging normally from the left ($z<0$) on a crystal, consisting of $N=4$ periods, is shown in Fig. 3. The results were obtained by the standard transfer matrix method, assuming for simplicity that $n_{\mathrm{in}}=n_{\mathrm{ex}}=n_1$. For such a sawtooth crystal, the transmittance is defined by three parameters: $kn_{\mathrm{av}}d, r_{21}$, and the number of periods *N*.

## Floquet–Bloch solutions within a sawtooth potential

In this section we analyze the behavior of the Floquet–Bloch solutions in the various regions of a sawtooth photonic crystal band structure depicted in Fig. 2. Three points, with $kn_{\mathrm{av}}d/\pi =1.115$ (point A), $kn_{\mathrm{av}}d/\pi =1.680$ (point B), $kn_{\mathrm{av}}d/\pi =1.831$ (point C), have been chosen to sequentially represent the bandgaps, allowed bands, and bandedges. All three points are characterized by the parameter $r_{21}=0.5$, which corresponds, for example, to a crystal with the refractive index increasing from $n_1=1.5$ to $n_2=4.5$. For such a crystal $n_{\mathrm{av}}=3.0$ and we take for simplicity a period of the crystal to be $d = 1\,\upmu \hbox {m}$.

At each of the above points (A, B, C), we construct both Floquet–Bloch functions (and a hybrid Floquet mode if necessary), using general representation of those functions in terms of the normalized solution, see Refs. Morozov and Sprung (2011), Morozov and Sprung (2012), as follows

16 $$\begin{aligned} F_{1,2}(z) & = u(z)+\frac{\rho _{1,2}-u(d)}{v(d)}\,v(z), \quad \text{ if } \,\, v(d)\ne 0, \nonumber \\ F_{1,2}(z) & = \frac{\rho _{1,2}-v'(d)}{u'(d)}\,u(z)+v(z), \quad \text{ if } \,\, u'(d)\ne 0. \end{aligned}$$

As previously mentioned, $u'(d)$ and *v*(*d*) are never simultaneously zero for a sawtooth crystal, so one of the above recipes always works. At the bandedges $F_2(z)=F_1(z)\equiv F(z)$ [$\cos (\varphi )=\pm 1$ and $u'(d)$ and *v*(*d*) are not simultaneously zero], and a second linear independent solution of Hill’s equation (2) is taken in the form of a hybrid Floquet mode

17 $$\begin{aligned} G(z) & = \frac{\rho \,d}{v(d)}\,v(z), \;\quad\text{ if } \, v(d)\ne 0, \nonumber \\ G(z) & = \frac{\rho \,d}{u'(d)}\,u(z), \quad \text{ if } \, u'(d)\ne 0. \end{aligned}$$

The corresponding total field *E*(*z*) generated by an optical wave of vacuum wavenumber *k* impinging on the crystal from $z<0$ is

18 $$\begin{aligned} E(z) & = \exp (ikn_{\mathrm{in}}z) + r \exp (-ikn_{\mathrm{in}}z), \quad z<0, \nonumber \\ E(z) & = C_1F_1(z) + C_2F_2(z), \quad 0<z<Nd, \nonumber \\ E(z) & = t\exp (ikn_{\mathrm{ex}}z), \quad z>Nd, \end{aligned}$$

where *r* and *t* are the amplitude reflection and transmission coefficients related to reflectance and transmittance as $R=|r|^2, T=n_{\mathrm{ex}}/n_{\mathrm{in}}\,|t|^2$. At the bandedges $F_2(z)=F_1(z)\equiv F(z)$, and one has to replace $F_2(z)$ with *G*(*z*). Finally, all of the above four constants $r, t, C_1, C_2$ are obtained from the boundary conditions at $z=0$ and $z=Nd$, where *N* is the number of periods.

**Fig. 4:**
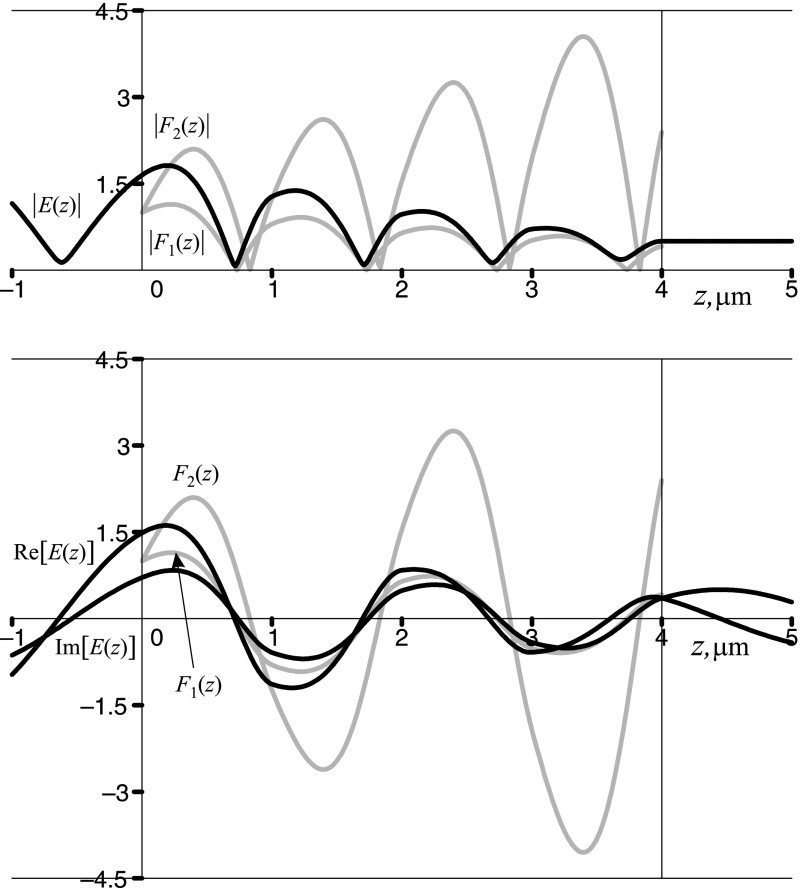
The moduli (*upper panel*) and the real and imaginary parts (*lower panel*) of the Floquet–Bloch solutions $F_{1}(z)$ and $F_{2}(z)$ (*grey lines*) and the overall field *E*(*z*) (*black lines*) at the point A located within the first bandgap of a sawtooth photonic crystal with $n_1=1.5, n_2=4.5, d = 1\,\upmu \hbox {m}, N=4$. Note that in the bandgaps ${\mathfrak {R}}[F_{1,2}(z)]=F_{1,2}(z), {\mathfrak {I}}[F_{1,2}(z)]=0$. The overall field *E*(*z*) is given by Eq. (18) and is shown here for the case $n_{\mathrm{in}}=n_{\mathrm{ex}}=n_1$

We now begin our analysis with the point A, located within the first bandgap of the crystal. The Floquet–Bloch solutions and the resultant field *E*(*z*) are shown in Fig. 4. As expected, the Bloch phase is complex since $\cos (\varphi ) = f(1.115\,\pi ,0.5) = -1.024$. Both Floquet multipliers are real-valued numbers, $\rho _{1} \approx -0.803$ and $\rho _{2}\approx -1.245$. The first Floquet–Bloch solution $F_{1}(z)$ decays along the axis of propagation, while the second one $F_{2}(z)$ grows in the same direction. The overall field *E*(*z*), which is a proper superposition of $F_1(z)$ and $F_2(z)$, also decays along the axis of propagation.

**Fig. 5:**
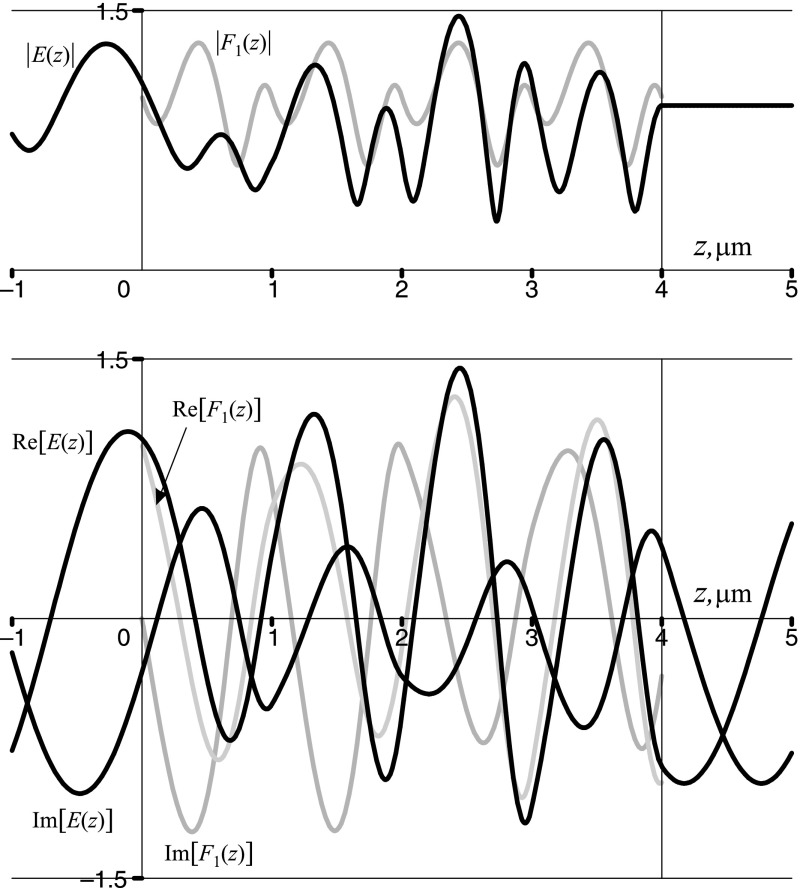
The moduli (*upper panel*) and the real and imaginary parts (*lower panel*) of the Floquet–Bloch solution $F_{1}(z)$ (*grey lines*) and the overall field *E*(*z*) (*black lines*) at the point B located within the second allowed band of a sawtooth photonic crystal with $n_1=1.5, n_2=4.5, d = 1\,\upmu \hbox {m}, N=4$. Note that in the allowed bands ${\mathfrak {R}}[F_2(z)]={\mathfrak {R}}[F_1(z)], {\mathfrak {I}}[F_2(z)]=-{\mathfrak {I}}[F_1(z)]$. The overall field *E*(*z*) is given by Eq. (18) and is shown here for the case $n_{\mathrm{in}}=n_{\mathrm{ex}}=n_1$

Point B is located within the second allowed band of the crystal, where, in particular, $\cos (\varphi ) = f(1.680\,\pi ,0.5) \cong 0.646$. The Floquet multipliers are complex valued numbers, $\rho _{1,2} \cong 0.646 \pm 0.763\,i$. Both the Floquet–Bloch solutions, which are complex conjugates of each other, as well as the total field are oscillating functions. The results are shown in Fig. 5.

**Fig. 6:**
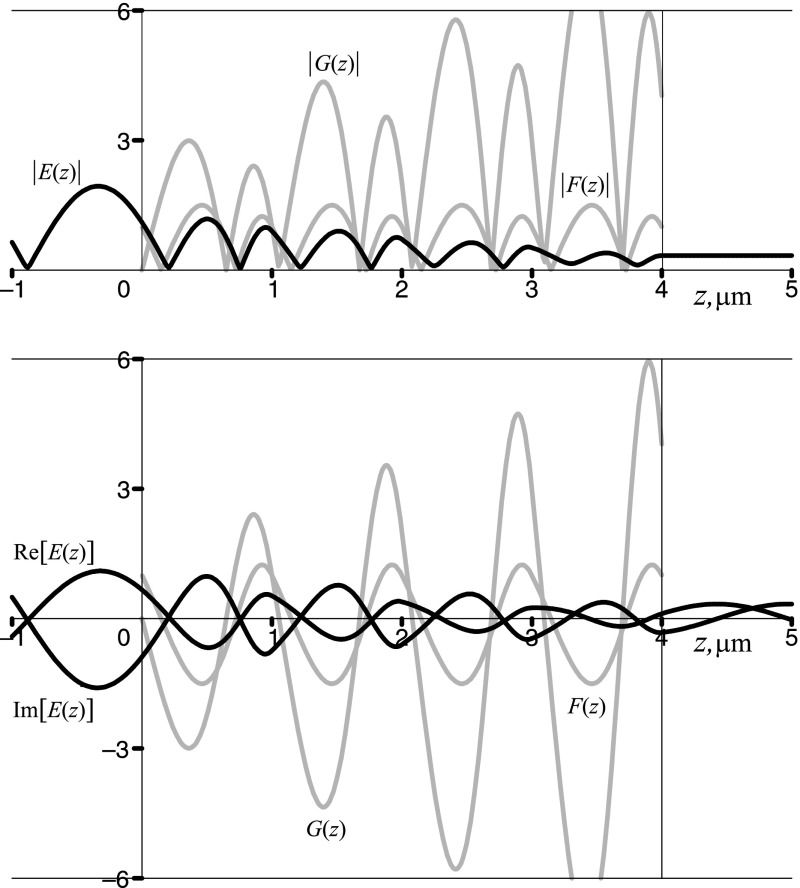
The moduli (*upper panel*) and the real and imaginary parts (*lower panel*) of the Floquet–Bloch periodic solution *F*(*z*) and the hybrid Floquet mode *G*(*z*) (*grey lines*), and the overall field *E*(*z*) (*black lines*) at the point C, located at the bandedge between the second allowed band and the second bandgap of a sawtooth photonic crystal with $n_1=1.5, n_2=4.5, d = 1\,\upmu \hbox {m}, N=4$. Note that ${\mathfrak {R}}[F(z)]=F(z), {\mathfrak {R}}[G(z)]=G(z), {\mathfrak {I}}[F(z)]={\mathfrak {I}}[G(z)]=0$. The overall field *E*(*z*) is given by Eq. (18) and is shown here for the case $n_{\mathrm{in}}=n_{\mathrm{ex}}=n_1$

Point C is located on the bandedge between the second allowed band and the second bandgap of the crystal, where $\cos (\varphi ) = f(1.831\,\pi ,0.5) = 1$. The Floquet multipliers coincide with each other, $\rho _{2} = \rho _{1} \equiv \rho =1$, and so do the Floquet–Bloch solutions, $F_2(z)=F_1(z) \equiv F_(z)$, which become periodic functions of period *d*. The hybrid Floquet mode *G*(*z*) is then constructed as a second linear independent solution of Hill’s equation (2). It grows within the crystal while the overall field *E*(*z*), which is a proper superposition of *F*(*z*) and *G*(*z*), decays within the crystal. The results are shown in Fig. 6.

## Conclusions

We found that the band structure of a 1D sawtooth photonic crystal in the case of normal light propagation is defined by two parameters only: the Fresnel reflection coefficient $r_{21}$ between the highest and the lowest refractive indices inside the crystal, and a period-average dimensionless wavenumber $kn_{\mathrm{av}}d$ of the light inside the crystal. To a good approximation the bandgaps have the same width, which is useful for applications where significant reflection is required in several frequency ranges. A typical behavior of the Floquet–Bloch solutions was illustrated for each characteristic region of the band structure, including the bandgaps, allowed bands, and bandedges. The absence of vanishing gaps (incipient bands) hinted in Mogilner and Loly (1992) was confirmed. This paper extends our understanding of light propagation through a sawtooth photonic crystal and adds further insight into the theory of periodic potentials in general.
